# Metastatic squamous cell carcinoma of the skin with clinical response to lapatinib

**DOI:** 10.1186/s40164-018-0111-z

**Published:** 2018-08-28

**Authors:** John D. Strickley, Aaron C. Spalding, M. Tye Haeberle, Timothy Brown, Don A. Stevens, Jae Jung

**Affiliations:** 1 University of Louisville School of Medicine, 323 E Chestnut St, Louisville, KY 40202 USA; 2 Norton Cancer Institute, 315 E. Broadway, Louisville, KY 40202 USA; 3 Division of Dermatology, University of Louisville School of Medicine, 3810 Springhurst Blvd, Ste. 200, Louisville, KY 40241 USA

**Keywords:** Metastatic cutaneous squamous cell carcinoma, Lapatinib, ERBB3, Next generation sequencing

## Abstract

**Background:**

Lapatinib is a tyrosine kinase inhibitor that blocks the HER2 receptor and is typically used in the setting of metastatic breast cancer. Both ERBB2 (HER2) and ERBB3 (HER3) belong to the same family of receptor tyrosine kinases. Dimerization of these receptors leads to activation of cell proliferation and survival pathways, granting oncogenic potential to dysregulated ERBB/HER receptors. Next generation sequencing (NGS) of tumors has ushered in a new era of personalized oncology therapy and has the ability to detect mutations in ERBB receptors.

**Case presentation:**

We present a patient with metastatic cutaneous squamous cell carcinoma who failed surgery, radiation, and anti-PD1 therapy, but showed clinical response to a drug targeting an ERBB3 mutation identified with NGS. Following initiation of the drug lapatinib, this patient exhibited dramatic tumor regression in the skin, soft tissue, bone and nerves.

**Conclusions:**

Cutaneous squamous cell carcinoma is the 2nd most common skin cancer in humans and future investigation of ERBB2 targeted therapies may provide an effective treatment strategy for patients with mutations in the ERBB2/3 pathway.

## Background

Lapatinib is a tyrosine kinase inhibitor used in the setting of ERBB2/HER2 positive metastatic breast cancer [1]. ERBB3 (aka HER3) resides within the same family of receptor tyrosine kinases. In the skin, this family of receptors play a major role in the regulation of keratinocyte proliferation and differentiation, thus have significant oncogenic potential [2]. Here we describe a case of cutaneous metastatic squamous cell carcinoma with an ERBB3 mutation that showed significant improvement in response to lapatinib.

## Case presentation

**Fig. 1:**
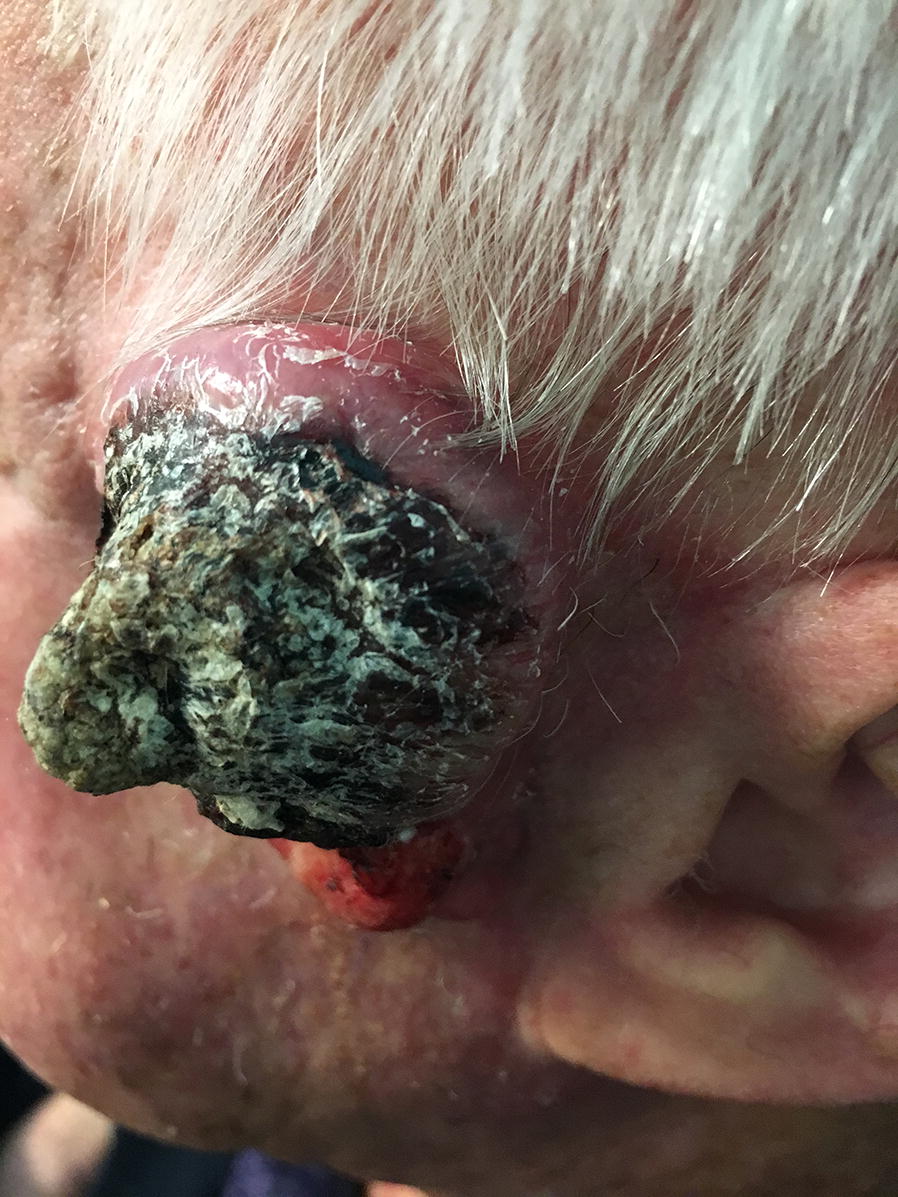
Clinical appearance of the recurrent squamous cell carcinoma of the left temple with zygomatic bone metastasis

A man in his 80s presented with 2 years of recurrent cutaneous squamous cell carcinoma of the left temple (Fig. 1) with zygomatic bone metastasis. He also had significant unilateral hearing loss secondary to perineural involvement. The 2 years of therapy preceding evaluation in our oncodermatology clinic is described below. In addition to Mohs micrographic surgery, the patient had also received two rounds of adjuvant radiotherapy. In the first round of radiotherapy, the patient received a total dose of 5000 cGy in 25 fractions delivered with 3D conformation irradiation to the tumor bed and facial nodal basins. Eight months later, a bony metastasis of the mandible led to another 5000 cGy dose, which was delivered in 25 fractions using intensity-modulated irradiation tracking along the V2 branch of the trigeminal nerve to the ipsilateral skull base and encompassing the cavernous sinus. Yet another bony metastasis was discovered 5 months later, at which time he consented to 5 cycles of off-label, palliative, compassionate-use nivolumab monotherapy. However, following 2 months of nivolumab treatment, repeat MRI showed continued tumor progression. At this time he presented to our clinic complaining of a 3 week history of a rapidly enlarging painful nodule over his left zygoma. Tumor genomic analysis of the nodule using next-generation sequencing (FoundationOne^®^, Cambridge, MA) revealed a somatic missense (R135C) mutation in the ERBB3/HER3 gene, as well as multiple other mutations (Table 1) and a high tumor mutation burden (75 mutations per megabase).

**Table 1:** List of mutations identified in next-generation sequencing with potential treatments, adapted from the patient’s FoundationOne^®^ report

Mutation	Description	Potential treatments^a^
ERBB3	R135C	Lapatinib, trastuzumab, afatinib, pertuzumab, ado-trastuzumab emtansine
NF2	Q400	Everolimus, temsirolimus, FAK inhibitors, lapatinib, trametinib
CDKN2A	P16INK4a D84Y and p14ARF 98L	Abemaciclib, ribociclib, palbociclib
CARD11	R888C	NF-kB inhibitors
FAT1	C3738fs*12	
LRP1B	G4199E	
RUNX1T1	R394W	
TERT promoter	− 139_− 138 CC > TT	
TP53	R196*, R282W	AZD1775, APR-246, SGT-53, CHK1 inhibitor with irinotecan, kevetrin

In the FAT1 and TP53 mutations, the “*” denotes a mutation in a stop codon. In the FAT1 mutation, “fs” denotes a frameshift mutation. In the TERT promoter mutation, “−” denotes the affected nucleotide, “_” denotes the range of affected residues, and “>” denotes a substitution mutation

^a^ Not all listed treatments are FDA-approved and some are drawn from data found in preclinical and/or early clinical studies

**Fig. 2:**
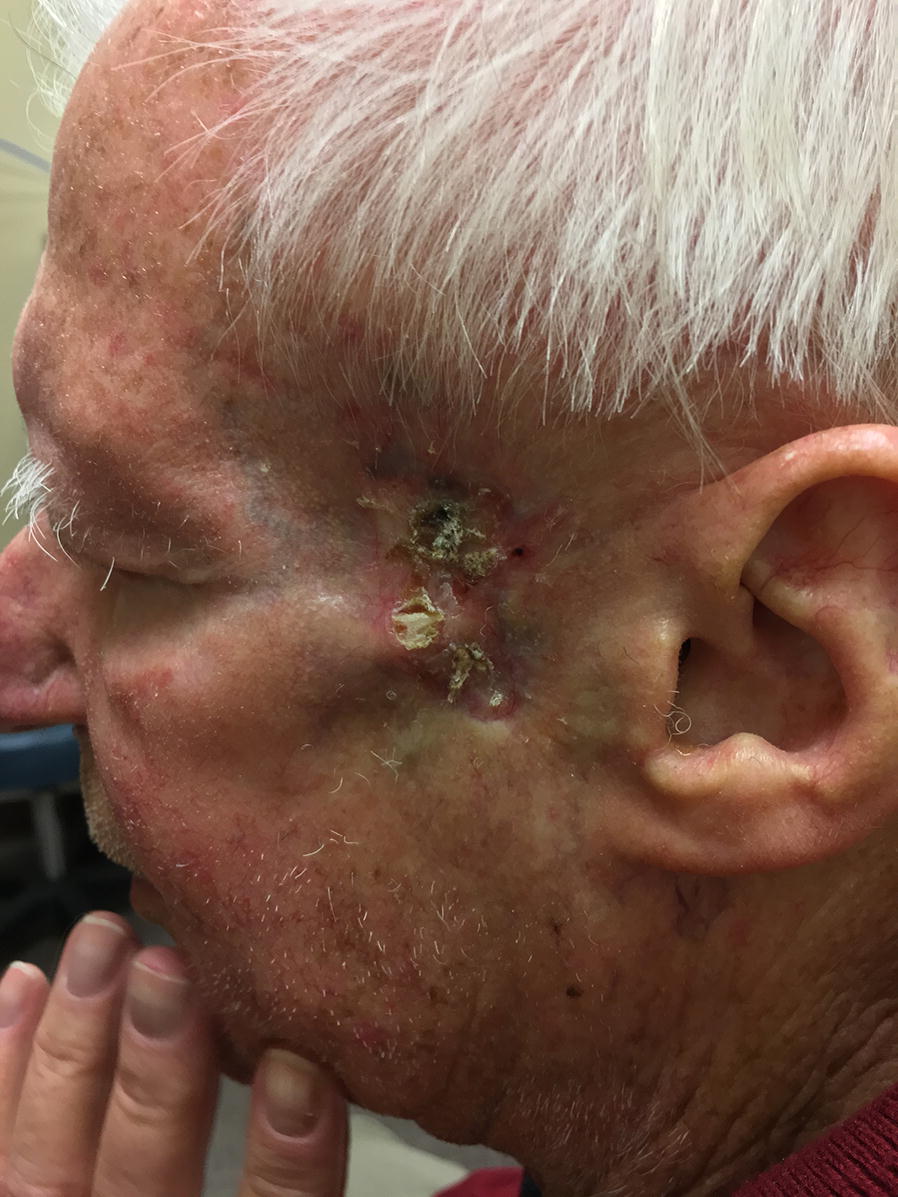
Clinical appearance of the tumor following surgical debulking and 6 months of lapatinib and nivolumab therapy

**Fig. 3:**
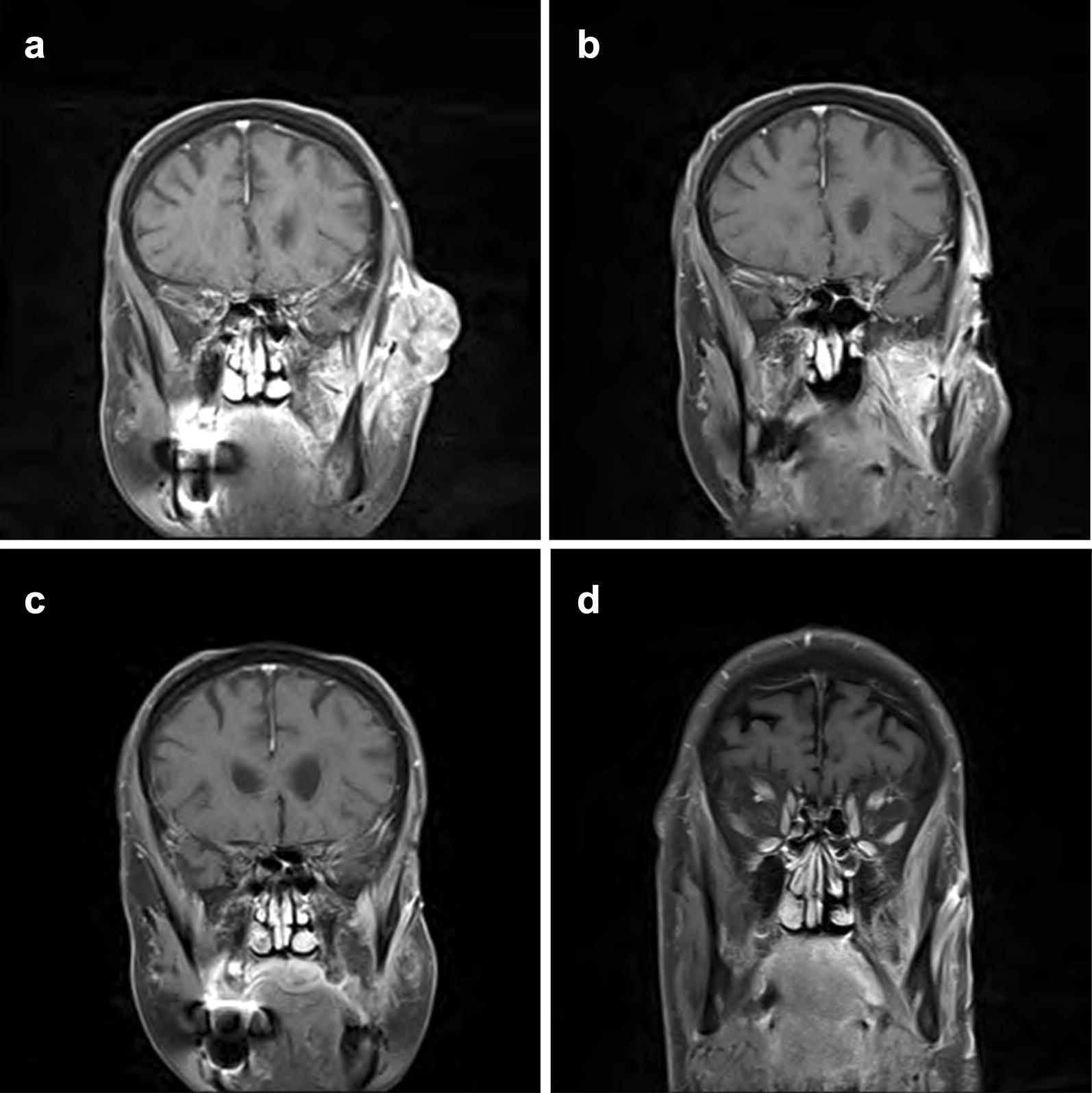
**a**–**d** Coronal T1+ Gadolinium MRI demonstrating 3.2 × 3.8 × 4.8 cm heterogeneous mass and progressive direct bone invasion extending to the junction of the left temporal bone and zygomatic arch. There is involvement of the deep left parotid gland at the level of the facial nerve with evidence of perineural extension along the V3 nerve to the foramen ovale. Figure 1b through 1d demonstrate dramatic improvement in the patient’s tumor burden

In an effort to target the ERBB3/HER3 mutation, therapy with 1,250 mg of lapatinib daily in combination with 240 mg nivolumab every 2 weeks was initiated. Additional tumor debulking in conjunction with cryotherapy to the base of the lesion was performed by our Mohs surgeon. Significant improvement in the clinical size of the lesion was noted after 2 months of lapatinib therapy. After 6 months, there was continued clinical improvement (Fig. 2) and MRI showed significant regression of muscle, nerve, and bone involvement (Fig. 3). The patient experienced a significant decrease in narcotic pain medication dependence and improvement in hearing of the ipsilateral ear. Other than fatigue, he experienced no side effects from this therapy.

## Discussion

This patient’s squamous cell carcinoma possessed a mutation in the ERBB3 (aka HER3) gene which encodes a receptor belonging to the human epidermal growth factor receptor (HER) family. Dysregulation of the HER family of receptors is most commonly implicated in breast cancer. Although studies of cancer involving the skin are limited, overexpression of ERBB3 has been demonstrated in squamous cell carcinoma, basal cell carcinoma, and melanoma [2, 3]. One preclinical study has shown that deletion of the ERBB3 gene provided significant skin cancer protection in mice, suggesting an important role for ERBB3 in skin carcinogenesis [2].

Signal transduction of HER receptors requires dimerization with another receptor within the family. ERBB3 has little ability to transduce signal via homodimerization, thus it relies on pairing with others, most notably ERBB2 (aka HER2). This combination of receptors has been found to be coexpressed more frequently in non-melanoma skin cancers when compared to normal skin [3]. The ERBB2 and ERBB3 heterodimer appears to be the most oncogenic dimeric combination [4]. The strength in this pairing lies in ERBB2′s potent stimulation of the mitogen-activated protein kinase (MAPK/ERK) pathway, and ERBB3′s stimulation of the phosphatidylinositol 3-kinase (PI3K) to AKT cell-survival pathway [5]. These findings suggest that this oncogenic heterodimer may be involved the carcinogenesis of skin cancers and could be a target of therapy.

Lapatinib is a dual tyrosine kinase inhibitor of ERBB1 (aka EGFR) and ERBB2 which is FDA-approved for the treatment of ERBB2 positive metastatic breast cancer. In vitro, this drug was shown to be more efficacious than other drugs (e.g. trastuzumab) when a given to cancer cells with ERBB3-activating somatic mutations, similar to the mutation described in this patient [6]. Our patient was given lapatinib which led to marked clinical improvement, exhibited by a significant decrease in tumor size, pain, and the return of his hearing.

Although the exact mechanism of this patient’s clinical response is unknown, we postulate that synergistic tumor destruction mechanisms enhanced the immune activation of anti-PD1. As in melanoma, it is likely that tumor recognition and destruction are dependent on the development and recognition of neo-antigens. Not all mechanisms that increase tumor destruction generate viable tumor antigens, for example, in a mouse model, cryotherapy with liquid nitrogen generated nearly 3 times as many tumor specific T cells as radiofrequency ablation [7, 8]. In our patient, we suspect that adjunctive measures, including tumor debulking surgery and liquid nitrogen cryotherapy, also contributed to his remarkable clinical response. Of note, it was difficult to identify a surgeon willing to perform debulking surgery for this patient even though debulking procedures are well known to provide palliation and augment systemic therapy in other tumor types [9].

Cutaneous SCC is the second most common malignancy and although surgery may be curative in local disease, effective therapy for metastatic disease remains elusive. The metastatic rate for primary tumors on sun-exposed skin is 5.2% and overall metastatic rate is 11%. Overall 5-year survival rate is 26.8% from all cutaneous sites [10]. Future study of HER2 targeted therapies may provide an effective treatment strategy for cutaneous squamous cell carcinoma patients with mutations in the HER2/3 pathway, including those with advanced disease.

## Abbreviations

NGS: next generation sequencing
HER: human epidermal growth factor receptor
MAPK/ERK: mitogen-activated protein kinase
PI3K: phosphatidylinositol 3-kinase
EGFR: epidermal growth factor receptor
